# Protein Phosphatase 2A Catalytic Subunit PP2A-1 Enhances Rice Resistance to Sheath Blight Disease

**DOI:** 10.3389/fgeed.2021.632136

**Published:** 2021-02-25

**Authors:** Qiu Jun Lin, Jin Chu, Vikranth Kumar, De Peng Yuan, Zhi Min Li, Qiong Mei, Yuan Hu Xuan

**Affiliations:** ^1^College of Plant Protection, Shenyang Agricultural University, Shenyang, China; ^2^Institute of Plant Protection, Liaoning Academy of Agricultural Sciences, Shenyang, China; ^3^Division of Applied Life Science (BK21 Program), Plant Molecular Biology and Biotechnology Research Center (PMBBRC), Gyeongsang National University, Jinju, South Korea

**Keywords:** PP2A-1, sheath blight, resistance, enhance, rice

## Abstract

Rice (*Oryza sativa*) production is damaged to a great extent by sheath blight disease (ShB). However, the defense mechanism in rice against this disease is largely unknown. Previous transcriptome analysis identified a significantly induced eukaryotic *protein phosphatase 2A catalytic subunit 1* (*PP2A-1*) after the inoculation of *Rhizoctonia solani*. Five genes encoding PP2A exist in rice genome, and these five genes are ubiquitously expressed in different tissues and stages. Inoculation of *R. solani* showed that the genome edited *pp2a-1* mutants using the CRISPR/Cas9 were more susceptible to ShB than the wild-type control, but other *PP2A* gene mutants exhibited similar response to ShB compared to wild-type plants. In parallel, *PP2A-1* expression level was higher in the activation tagging line, and *PP2A-1* overexpression inhibited plant height and promoted the resistance to ShB. PP2A-1-GFP was localized in the cytoplasm and nucleus. In addition, *R. solani*-dependent induction kinetics of pathogen-related genes *PBZ1* and *PR1b* was lower in *pp2a-1* mutants but higher in *PP2A-1* activation line compared to those in the wild-type. In conclusion, our analysis shows that PP2A-1 is a member of protein phosphatase, which regulates rice resistance to ShB. This result broadens the understanding of the defense mechanism against ShB and provides a potential target for rice breeding for disease resistance.

## Introduction

*R. solani* is the causative agent of ShB in rice (Savary et al., 1995; Suryadi et al., 2013), and which damages rice during the entire growth period, and predominantly targets the leaves, sheaths, and panicles, eventually resulting in the withering and lodging of the entire plant. A severe form of ShB can lower the rice produce by ~50% (Savary et al., 2000). The rapid variation, wide host range, and high survival ability of the pathogen can make the disease control more challenging (Taheri and Tarighi, 2011; Yellareddygari et al., 2014; Singh et al., 2019). Currently, there is a dearth of ShB-resistant cultivars, therefore, the strategies to quell ShB involve the use of fungicides (Savary et al., 2000). However, fungicides directly affect the living environment of other microorganisms and increase the cost of cultivation. Thus, to develop ShB resistance in rice, it is necessary to isolate resistant cultivars and understand their underlying defense mechanisms against ShB.

Extensive studies have been performed to investigate the mechanism of rice defense against ShB. Overexpression of chitinase, β-1,3-glucanase, or OsPGIP1 (polygalacturonase-inhibiting protein) (Shah et al., 2009; Mao et al., 2014; Zhu et al., 2019), OsACS2 (key enzyme in ethylene synthesis) (Helliwell et al., 2013), OsGSTU5 (tau class glutathione-*S*-transferase 5) (Tiwari et al., 2020), and Os2H16 (Li et al., 2013, 2018) were found to promote rice resistance to ShB. In addition, *BSR2* (*broad-spectrum resistance 2*) (Maeda et al., 2019) or a transcription factor complex including LPA1 (indeterminate domain 14, IDD14) and IDD13 (Sun et al., 2019, 2020) were reported to positively regulate rice resistance to ShB while *SWEET11* (*sugar will eventually be exported transporter 11*) (Gao et al., 2018) exhibited a negative regulation. The transcription factor OsWRKYs also plays an important role in resistance to sheath blight (Peng et al., 2012, 2016; Wang et al., 2015; Jimmy and Babu, 2019; Yuan et al., 2020). In addition, salicylic acid-dependent immunity showed a positive regulation in ShB resistance in rice and *Brachypodium distachyon* (Kouzai et al., 2018).

Protein phosphatase also plays an important role in plant defense response. The protein phosphatases (PPs) with a vast array of structures and functions are mainly categorized as serine/threonine (Ser/Thr) PPs and protein tyrosine phosphatases (PTPs). PP1, PP2A, PP2B, and PP2C account for the sub-divisions of the protein tyrosine phosphatase group. The PP2A complex comprises three subunits: A, B, and C with scaffolding, regulatory, and catalytic roles, respectively (Yu et al., 2005; Durian et al., 2016). The role of PP2A protein in plant abiotic stress signal transduction has been confirmed. For instance, drought and elevated salinity induce high levels of *OsPP2A-1* and *OsPP2A-*3, the closely associated genes coding for the C-subunit of PP2A (Yu et al., 2003). In *Arabidopsis*, the growth of roots and shoots is augmented by *PP2A-C5* overexpression in the presence of several salts indicating the vital function of protein in growth to combat salinity (Hu et al., 2017). *AtPP2A* is involved in acclimation to light as well as when responding to pathogens, both based on the regulation of ROS (Rahikainen et al., 2016; Máthé et al., 2019). Exposure of wheat to *R. cerealis* or hydrogen peroxide showed elevated *TaPP2Ac-4B* and *TaPP2Ac-4D* RNA levels revealing the involvement of PP2A in the biotic stress response. Silencing of *TaPP2A* in wheat boosted the expression of ROS-scavenging and pathogenesis-related (PR) RNA molecules (Zhu et al., 2018). Resistance to *Botrytis cinerea* and leaf senescence in *Arabidopsis* involves the role of PP2A-B′γ. The swift induction of the gene coding for the heterotrimeric PP2A catalytic subunit, *LePP2A-1* was observed when resistant tomato plants were challenged with *Pseudomonas syringae* pv. *tomato* (a virulent strain) (He et al., 2004). A mutation which was isolated from rice blast fungus was inserted into the promoter region of MoPPG1, a ser/thr-PP2A catalytic subunit (PP2Ac) gene, which made the mutant defective in the growth of vegetative mycelium and could not cause disease (Du et al., 2013). *Fusarium graminearum* contains three kinds of PP2A (FgPp2A, FgSit4, and FgPg1), which play a key role in the growth, development, and pathogenicity of fungi (Liu et al., 2018). Our recent transcriptomic study showed the sensitivity of *PP2A-1* expression to *R. solani* infection (Yuan et al., 2020). However, PP2A function in rice defense to ShB is unknown.

In this study, *PP2A-1* was significantly induced following *R. solani* inoculation. Further bioinformatics, genetic, and molecular analyses were performed to identify the function of PP2A family members in rice defense to ShB. Our results broaden the knowledge of the underlying ShB defense mechanisms and provide a potential target for resistant breeding in rice.

## Materials and Methods

### Plant and Fungal Materials

Four rice lines/cultivars, including Japonica rice cultivar Dongjin (DJ), Zhonghua11 (ZH11), *pp2a-1* CRISPR/Cas-9 genome editing mutants in ZH11 background, and *PP2A-1* activation tagging line (*PP2A-1 OX*) in DJ background were used in this study. All the rice lines used in this study were grown in a greenhouse in natural light. The type strain used in this study was *R. solani* AG1-IA.

### Construction of the CRISPR/Cas9 Plasmids

**Figure 1:**
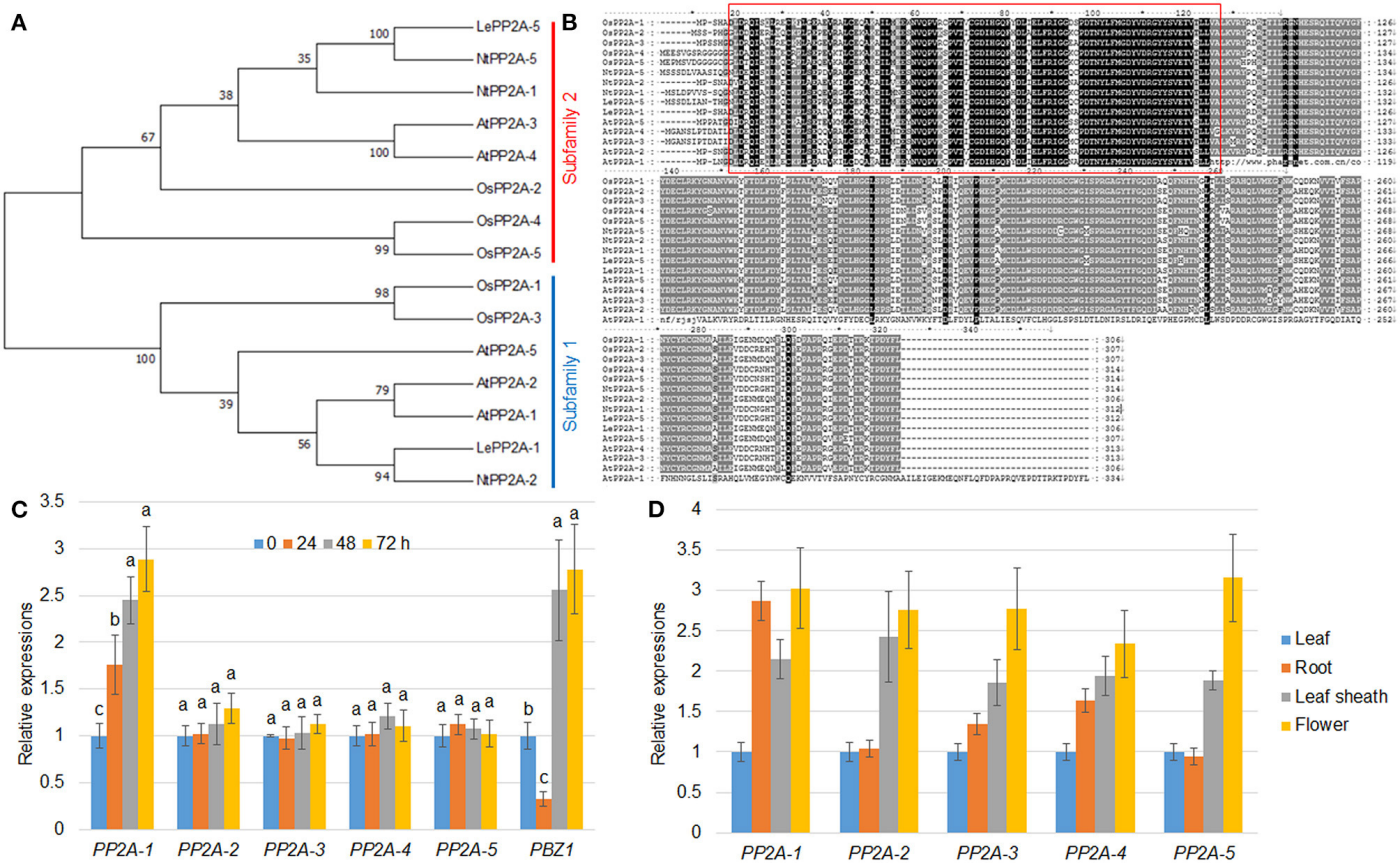
Phylogenetic and expression pattern analysis. **(A)** The maximum likelihood was utilized for the phylogenetic tree construction. The phylogenetic tree was generated by MEGA 7.0 on the basis of PP2A amino acid sequences from *Oryza sativa* (OsPP2As), *Arabidopsis thaliana* (AtPP2As), *Solanum lycopersicum* (LePP2As), and *Nicotiana tabacum* (NtPP2As). The PP2As were classified into subfamily 1 and 2 that were marked with blue and red, respectively. **(B)** The protein sequence identity of the PP2As listed in **(A)** was analyzed by the Clustal W program. The red box indicates a highly conserved region between PP2As. **(C)** *R. solani* inoculation-mediated *PP2A* expression. The five *PP2A* and *PBZ1* gene expression patterns were evaluated after 0, 24, 48, and 72 h following *R. solani* inoculation. Normalization of expression was done with reference to the internal control *ubiquitin*. The experiments were done in triplicate. Different letters indicate significant differences at *P* < *0.05*. **(D)** Tissue-specific expression patterns of five *PP2A* genes were analyzed using the RNA extracted from the leaf, root, leaf sheath, and flower. *Ubiquitin* was used as the internal control to normalize expression levels. The experiments were done in triplicate.

The human codon-optimized hSpCas9 (Cong et al., 2013) was linked to the maize ubiquitin promoter (UBI) in an intermediate plasmid followed by its insertion into a binary pCAMBIA1300 vector (Cambia, Australia) harboring the *HPT* (*hygromycin B phosphotransferase*) gene. A point mutation kit (Transgen, China) was used to eliminate the original *Bsa*I site in the backbone of pCAMBIA1300. A OsU6 promoter fragment (Feng et al., 2013), *ccdB*, a gene for negative selection flanked by two *Bsa*I sites, and a pX260- derived sgRNA (Cong et al., 2013) were inserted employing an In-Fusion cloning kit (Takara, Japan) into this vector to produce the CRISPR/Cas9 binary vector pBGK032 (Figure 1). The vector was maintained in *Escherichia coli* strain DB3.1.

The targeting specificity and the selection of the 23-bp targeting sequences (including PAM) was done employing a BLAST search (http://blast.ncbi.nlm.nih.gov/Blast.cgi) against the rice genome (Hsu et al., 2013). The designed targeting sequences were annealed to synthesize the oligo adaptors. The vector pBGK032 was restriction digested using *Bsa*I and purified employing a DNA purification kit (Tiangen, China). This was ligated with 0.05 mM of oligo adaptor (10 μL) resulting in CRISPR/Cas9 plasmids, which were directly transformed in competent *E. coli* cells.

### Transformation of Plants and Mutation Detection

*Agrobacterium tumefaciens* strain EHA105 was transformed with the CRISPR/Cas9 plasmids followed by rice transformation following an earlier published protocol (Nishimura et al., 2006). The genomic DNA from these transformants was extracted, and PCR was conducted employing primer pairs flanking the designed target site. The Degenerate Sequence Decoding approach was applied to directly sequence and identified the PCR products (300–500 bp) method (Ma et al., 2015).

### RNA Extractions

The total cellular RNA was extracted from the 1-month-old plant sheath, roots, leaves, or 3-month-old flower tissues. For analysis of *R. solani*-mediated gene expression, Trizol reagent (Invitrogen, China) was used to isolate the total RNA from 0.1 g of rice leaves, roots, leaf sheath, and flowers. Then the product was purified using the RNeasy mini kit (Promega, China) and RNase-Free DNase I (Promega, China) following manufacturer's instructions. The cDNAs were synthesized with M-MLV Reverse Transcriptase (Promega) kit following manufacturer's instructions.

### Sequence Analysis of PP2A

The PP2A amino acid sequences were isolated from *Arabidopsis*, rice, tobacco, and tomato to perform phylogenetic analysis. MEGA7 software was used for multiple sequence alignment of the original sequences. The comparison results were edited by GeneDoc to export the multi-sequence alignment results graph. MEGA7 software was used for phylogenetic tree construction using the nearest neighbor-joining method (Kumar et al., 2016).

### cDNA Synthesis and qRT–PCR

Reverse transcription using 2 μg of each purified RNA sample was done using a Prime Script TMRT Reagent Kit with gDNA Eraser (TaKaRa, China) in accordance with the provided instructions. qRT-PCR was performed on the ABI 7500 RT-PCR system (Applied Biosystems, United States). The composition of the mix was: 10 μL 2× SYBR Premix Ex Taq, 0.4 μL 50× ROX Reference Dye II, 0.4 μM of each primer, and 5 μL of the cDNA template (50-fold dilution) in a net volume of 20 μL. The conditions were: 95°C for 30 s; 95°C for 5 s, 58°C for 15 s, and 72°C for 34 s for 40 cycles. The 2^[−*DeltaDeltaC*(*T*)]^ approach was employed to estimate the expression levels of target gene(s) relative (Livak and Schmittgen, 2001). *Ubiquitin* was used as an internal reference. Table 1 presents the primers in this study.

**Table 1:** Sequence of the primers used in this study.

**Primer**	**Sequence**
Ubiquitin F	CACGGTTCAACAACATCCAG
Ubiquitin R	TGAAGACCCTGACTGGGAAG
PP2A-1 F	CACGGTGTTCAGCGCCCCAAAC
PP2A-1 R	CGCGTTGTGTCCGGCTCTATTTG
PP2A-2 F	GCTAGAGCTCACCAGTTGGTCATG
PP2A-2 R	TACATCTGGCTCTCCCCTTCTTG
PP2A-3 F	CTCTCATCTCAAGGGCACATCAAC
PP2A-3 R	TGTGTCTGGTTCAATTTGCCGAGGAG
PP2A-4 F	CGAACAAAAGGTCGTGACCATATTC
PP2A-4 R	ATCAGGTGTTCTCCGTGTCACATC
PP2A-5 F	TAGCTCGGGCTCATCAACTAGTTATG
PP2A-5 R	AAATAATCGGGCGTCCTCCGTGTCAC
PBZ1 F	CCCTGCCGAATACGCCTAA
PBZ1 R	CTCAAACGCCACGAGAATTTG
PR1b F	GCGTCTTCATCACATGCAACTA
PR1b R	ACCTGAAACAGAAAGAAACAGAGG
PP2A-1 GFP F	CCATGGATGCCGTCGCACGCGGATCTGGAC
PP2A-1 GFP R	AGATCTCAAAAAGTAGTCGGGGGTCTTGCGC

### Inoculation With *R. solani* and Scoring Response of Rice Plants

Rice plants were grown in the glasshouse for 1 month prior to inoculation with the pathogen *R. solani* AG1-IA. The second leaf of the main tiller was cut into 10-cm slices, placed on wet filter paper and stored in a culture dish (36 × 36 × 2.5 cm). In a completely randomized design, five leaves were placed in each plate, with a total of three replicate plates for each treatment. The fungal plug (7 mm in diameter) was cut from the Potato Dextrose Agar (PDA) plate with *R. solani* and placed on the back of the leaf. The leaves were cultured for 72 h at 25°C under continuous light, and the moisture of the filter paper was maintained with sterile water (Gao et al., 2018). Measurement from 0 (no lesion) to 9 (lesions occupying 90–100% of the leaf surface) was done after visual observation. Scores from one to eight represented 10–80% diseased leaf area (Prasad and Eizenga, 2008).

Rice plants that were cultured in a greenhouse for 1 month prior to the tillering stage were used for inoculation. The sheath of the first leaf of the main stem was inoculated with *R. solani* AG1-IA. The PDA fungal plug was inoculated into rice leaf, sprayed with sterile water, and the severity of the disease was determined after 24, 48, and 72 h.

### Construction of PP2A-1-GFP Plasmid and Its Subcellular Localization

*PP2A-1* ORF region was amplified by PCR and moved into pCAMBIA1302 vector to create *PP2A-1-GFP* plasmid. The *Agrobacterium*-mediated transient expression approach was followed to introduce the fusion proteins into *Nicotiana benthamiana* (Kim et al., 2009). The location of the protein was monitored via GFP fluorescence with a confocal microscope (SP5; Leica, Solms, Germany).

### Statistical Analysis

The significant differences between different groups were analyzed using Microsoft Excel to compute the mean, standard deviation, and the Student's *t*-test. Dunnett's test was done employing the SPSS 19.0 statistical software.

## Results

### Inoculation of *R. solani* Significantly Induced *PP2A-1* Expression

Our previous transcriptome analysis identified that *PP2A-1* expression was induced by the inoculation of *R. solani* AG1-IA (Yuan et al., 2020). Rice genome harbors five PP2A isoforms, and a phylogenetic analysis of PP2A proteins from *Arabidopsis*, rice, tobacco, and tomato revealed that OsPP2A-1 clustered with OsPP2A-3, NtPP2A-2, AtPP2A-1, AtPP2A-2, AtPP2A-5, and LePP2A-1, all of which belong to Subfamily II, while OsPP2A-5, OsPP2A-4, OsPP2A-2, AtPP2A-3, AtPP2A-4, NtPP2A-1, NtPP2A-5, and LePP2A-5 belong to Subfamily I (Figure 1A). The homologous sequence alignment of PP2As showed that OsPP2A-1 and OsPP2A-3 shared 98% similarity. The red box indicates a highly conserved region between the five PP2As (Figure 1B). qRT-PCR of the 5 *PP2A* genes was done for the verification of the transcriptome data post-inoculation with *R. solani* after 0, 24, 48, and 72 h. The results indicated that only *PP2A-1* expression was induced by *R. solani* infection, and *PP2A-1* expression was the highest 72 h after inoculation, while the other four *PP2A* genes did not respond to *R. solani*. *PBZ1*, a marker gene was used for evaluating pathogen infection, its expression was down-regulated at 24 h after inoculation, while it was up-regulated after 48 h and 72 h of inoculation (Figure 1C; Table 2). In addition, tissue-specific expression of *PP2As* was examined by qRT-PCR. All *PP2As* were expressed in root, leaf sheath, leaf, and flower tissues, while *PP2A-1* was expressed highly in root and flower, indicating that *PP2As* were ubiquitously expressed in different tissues and developmental stages (Figure 1D).

**Table 2:** *R. solani* inoculation-mediated expression patterns of *PP2A* genes.

**Gene**	**Locus number**	**Description**	**Log_**2**_FC**	***P*-value**
*OsPP2A-1*	Os06g0574500	Protein Phosphatase 2A catalytic subunit 1	1.02878	0.00063
*OsPP2A-2*	Os03g0805300	Protein Phosphatase 2A catalytic subunit 2	N/A	N/A
*OsPP2A-3*	Os02g0217600	Protein Phosphatase 2A catalytic subunit 3	N/A	N/A
*OsPP2A-4*	Os10g0410600	Protein Phosphatase 2A catalytic subunit 4	N/A	N/A
*OsPP2A-5*	Os03g0167700	Protein Phosphatase 2A catalytic subunit 5	N/A	N/A

### *pp2a-1* Mutants Are Susceptible to ShB

**Figure 2:**
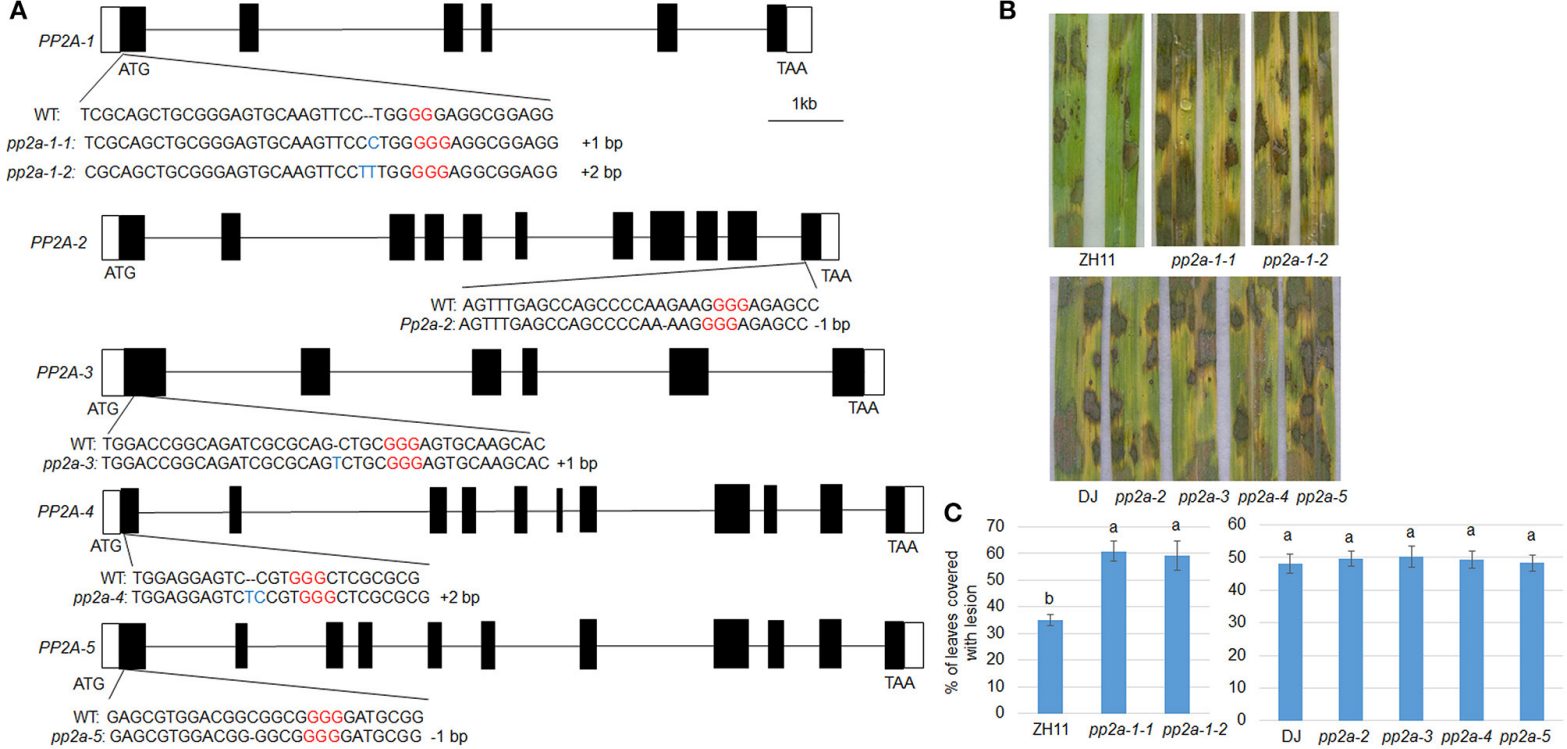
The genomic structure and defense response of *PP2As*. **(A)** Black and white boxes indicate the exon and UTR regions, respectively, while the lines indicate introns. The wild-type (WT) and CRISPR/Cas9-induced edited sequences are shown below the exon. **(B)** The leaves from the ZH11, *pp2a-1* (*#1, #2*), DJ, *pp2a-2, pp2a-3, pp2a-4*, and *pp2a-5* were inoculated with *R. solani* AG1-IA. **(C)** Shows the leaf surface lesion areas. Data show the average ± SE (*n* > 10). Different letters above the bars denote statistically significant differences (*P* < *0.05*).

To analyze the function of *PP2A* genes in rice defense to ShB, Crispr/Cas9 induced genome editing mutants for *PP2As* were generated. The *PP2A* genes consist of multiple exons and introns in the genome (Figure 2A). The sequencing of *PP2A* genome editing mutants revealed that *pp2a-1* mutants have a genomic lesion in the first exon with 1 or 2-bp insertions (*pp2a-1-1, pp2a-1-2*) and the *pp2a-2* mutant has a 1-bp deletion in the 11th exon. The *pp2a-3, pp2a-4*, and *pp2a-5* mutants contained edited sequenced in the first exon with a 1-bp insertion, 2-bp insertion, and 1-bp deletion, respectively (Figure 2A). After inoculation with *R. solani* AG1-IA, *pp2a-1* genome editing mutants in ZH11 background were more susceptible than ZH11 plants, showing obvious chlorosis (Figure 2B), while other *pp2a* genome editing mutants in DJ background had no obvious disease grade differences compared with that in wild-type plants (DJ) (Figure 2B). The lesion coverage on leaves of ZH11 (wild-type), *pp2a-1-1*, and *pp2a-1-2* were 34.1, 60.8, and 59.2%, respectively. However, the lesion area on DJ, *pp2a-2, pp2a-3, pp2a-4*, and *pp2a-5* were 48.2, 49.3, 50.1, 49.5, and 48.4%, respectively (Figure 2C).

### *PP2A-1* Overexpression Enhanced Rice Resistance to ShB

**Figure 3:**
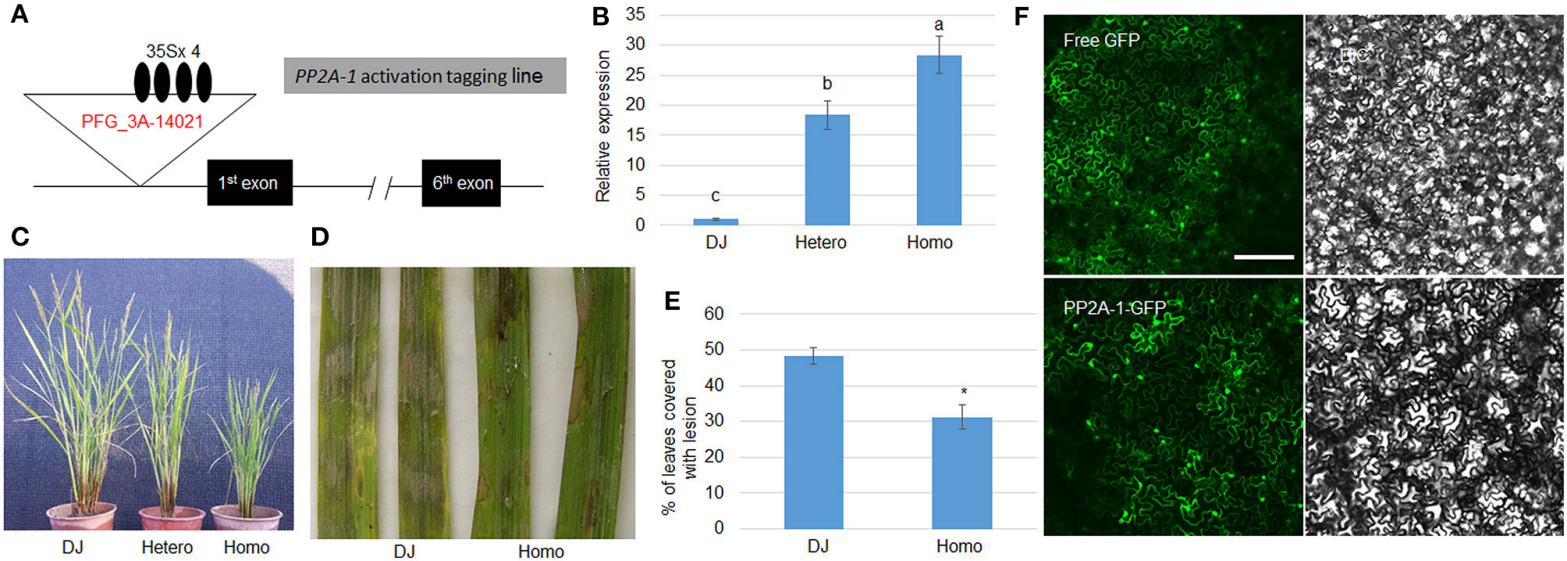
Genomic structure and defense response of *PP2A-1* overexpressor and PP2A-1 subcellular localization. **(A)** Genomic structure of *PP2A-1* activation tagging (*PP2A-1 OX*) line. Black boxes are demonstrative of exons and lines for introns. The T-DNA insertion site is shown as the white triangle and the black oval represents the 35S promoter. PFG_3A-14021 is the ID number of the mutant in the SALK database. **(B)** *PP2A-1* expression levels in DJ (wild-type), *PP2A-1* heterozygote, and homozygote plants. Normalization of expression was done with reference to the internal control *ubiquitin*. The experiments were done in triplicate. Significant changes are illustrated by various letters (*P* < *0.05*). **(C)** Three-month-old DJ, *PP2A-1* heterozygous, and homozygous plants. **(D)** Leaves from the DJ and *PP2A-1* homozygous plants were inoculated with *R. solani* AG1-IA. **(E)** The lesion areas on the leaf surfaces are shown in **(D)**. Data show the average ± SE (*n* > 10). Different letters above the bars denote statistically significant differences (*P* < *0.05*). **(F)** Free GFP and PP2A-1-GFP were expressed in tobacco leaves. GFP and bright-field channels Scale bar = 20 μm.

Since *pp2a-1* mutants were susceptible to ShB, the *PP2A-1* overexpression plants were further examined in response to ShB. We isolated a *PP2A-1* activation tagging line (*PP2A-1 OX*) in a T-DNA insertional library (Jeong et al., 2002). In the activation tagging line, T-DNA was inserted in the promoter region in which four copies of the 35S promoter activated the *PP2A-1* expression (Figure 3A). The qRT-PCR results indicated that *PP2A-1* expression was significantly higher in hetero- and homozygous *PP2A-1* activation tagging plants than wild type and the *PP2A-1* expression level was significantly higher in homozygous compared to heterozygous plants (Figure 3B). *PP2A-1 OX* lines displayed a semi-dwarf phenotype, with homozygous plants significantly shorter than heterozygous and wild-type plants (Figure 3C). Next, we selected the homozygous *PP2A-1 OX* to inoculate *R. solani* AG1-IA. The lesion area of *PP2A-1* homozygous overexpression plants was smaller than that of DJ (wild-type) after 48 h of inoculation (Figure 3D). The lesion coverage on leaves was 48.1 and 30.8%, respectively (Figure 3E), indicating that overexpression of *PP2A-1* enhanced rice resistance to ShB. In addition, PP2A-1-GFP and free GFP were expressed in tobacco leaves, and PP2A-1-GFP signal was detected in the cytoplasm and nucleus (Figure 3F).

### *PP2A-1* Positively Regulates Defense Gene Expression

**Figure 4:**
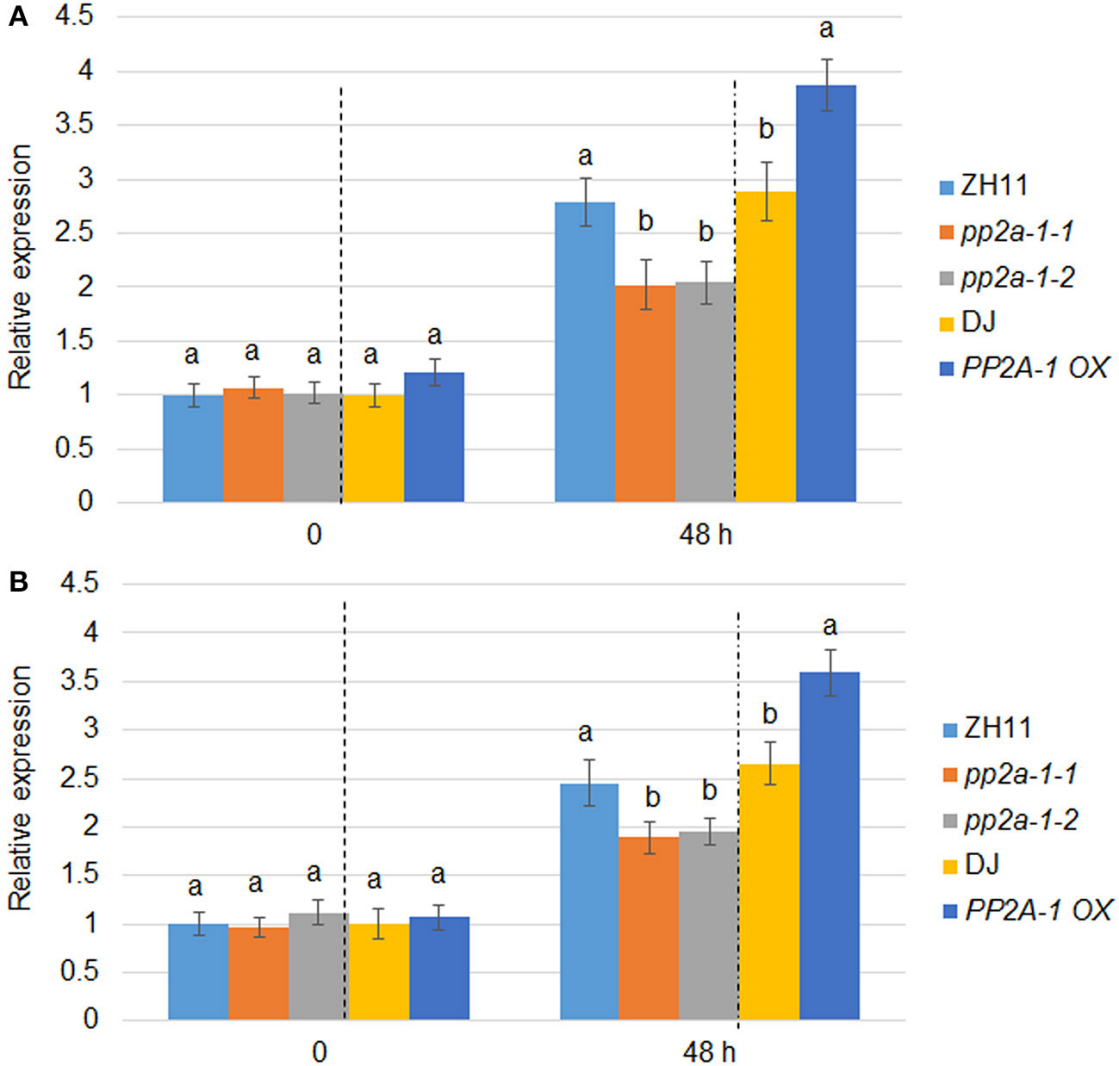
*PBZ1* and *PR1b* expression patterns in wild-type, *pp2a-1*, and *PP2A-1* overexpression plants. **(A)** *PBZ1* and **(B)** *PR1b* expression after *R. solani* inoculation. The *PBZ1* and *PR1b* gene expression patterns were evaluated after 0 and 48 h following *R. solani* inoculation. ZH11 is the control of *pp2a-1-1* and *pp2a-1-2*, while DJ is the control of *PP2A-1 OX* plants. Normalization of expression was done with reference to the internal control *ubiquitin*. The experiments were done in triplicate. Significant changes are illustrated by various letters (*P* < *0.05*).

*PP2A-1 OX* plants were less susceptible while *pp2a-1* mutants were more susceptible to ShB compared to the wild-type control. The expression patterns of defense genes *PBZ1* and *PR1b* in wild-type, *pp2a-1*, and *PP2A-1 OX* plants were examined following *R. solani* inoculation. qPCR results showed that there was no significant difference in the expression levels of *PBZ1* and *PR1b* among wild-type, genome editing mutants, and overexpression lines with no *R. solani* inoculation. However, the expression level of *PBZ1* in *pp2a-1* mutants was significantly lower than that in control ZH11, while it was higher in *PP2A-1 OX* plants than that in wild-type DJ after 48 h of inoculation (Figure 4A). The expression pattern of *PR1b* was similar to that of *PBZ1* at 48 h of inoculation, which showed lower and higher induction kinetics in *pp2a-1* mutants and *PP2A-1 OX* compared to that in wild-type plants, respectively (Figure 4B).

## Discussion

Rice ShB caused by *R. solani*, bacterial leaf blight caused by *Xanthomonas oryzae*, and rice blast caused by *Magnaporthe oryzae* are three major diseases that significantly affect rice yield in China (Suryadi et al., 2013). The use of resistant varieties has been the primary means of disease control. However, due to the lack of resistant varieties and that sclerotium has a strong reproductive capacity, ShB control is challenging. Therefore, it is necessary to screen disease resistance genes and understand their resistance mechanism. Previous studies showed that PP2A regulates the development of lateral and primary roots, seed germination, and abiotic stress response against high concentration of sugar, salt, and drought (Yu et al., 2003; País et al., 2009; Liu et al., 2014; Hu et al., 2017). PP2A also plays important roles in biotic stress resistance. For example, AtPP2A is involved in regulation of PTI (pathogen-associated molecular pattern-triggered immunity) upon response to *P. syringae* pv. *tomato* (Pto) DC3000 infection; *LePP2A* gene was rapidly induced by inoculating with the model strain *P. syringae* pv. *tomato; TaPP2A-4B* and *TaPP2A-4D* may negatively regulate wheat defense response to *R. cerealis* infection by triggering the generation of ROS and PTI-mediated induction of PR genes (He et al., 2004; Segonzac et al., 2014; Durian et al., 2016; Zhu et al., 2018), suggesting that PP2A may be a key regulator of PAMP induced immunity. In rice, the induction of okadaic acid-dependent AMY3 and RCht2 (rice chitinase) transcription are regulated via the PP2A signal transduction pathway (Luan et al., 1993; Kim et al., 1998). However, the role of PP2A in rice disease resistance still remains unclear.

Our results indicate that the protein phosphatase 2A catalytic subunit OsPP2A-1 enhances resistance to sheath blight disease in rice. In our previous transcriptome analysis, *OsPP2A-1* was significantly induced by *R. solani* inoculation (Yuan et al., 2020). The CRISPR/Cas9-mediated genome editing lines revealed higher susceptibility of *pp2a-1* mutants to ShB, compared to wild-type control and other *PP2A* mutants (Figure 2). While *PP2A-1* O*X* lines displayed a semi-dwarf phenotype, homozygous plants were significantly shorter than heterozygous and wild-type plants (Figure 3C). Inoculation of *R. solani* AG1-IA demonstrated that the *PP2A-1 OX* lines were less susceptible to ShB. The *PP2As* are ubiquitously expressed in different stages and tissues, and PP2A-1-GFP was localized at the cytosol and nucleus in tobacco leaves. The *PP2A-1* expression level was significantly higher in the activation tagging line, with higher *PP2A-1* expression inhibiting plant height while promoting ShB resistance.

The vital involvement of PP2A in responding to pathogens by plants has been demonstrated in recent studies (He et al., 2004; Zhu et al., 2018). For example, in *A. thaliana*, RLKs FLS2 (flagellin sensing receptor2) recognizes and EFR (EF-Tu receptor) is capable of recognizing the EF- Tu (elongation factor), both are PAMPs (pathogen-associated molecular pattern) of bacterial pathogens. The autophosphorylation and functioning of BAK1 (BRI1-associated kinase 1) is limited by PP2A-holoenzyme (Segonzac et al., 2014). *PP2A-c4* and *PP2A-a1* gene knockout mutants display a stronger resistance to virulent *P. syringae* pv. *tomato* DC3000 (Segonzac et al., 2014). The BSMV-VIGS (barley stripe mosaic virus–induced gene silencing) approach was applied to augment *R. cerealis* resistance in wheat attributed to *TaPP2Ac-4B* and *TaPP2Ac-4D* knock-down, suggesting the negative regulation of TaPP2A to wheat sharp eyespot. In many species, PP2A appears to function as a negative regulator, while OsPP2A-1 was found to positively regulate resistance to *R. solani* in this study. It seemed that the same gene may play different functions in response to infection of different types of pathogens. For example, WRKY transcription factors were more resistant to the hemibiotrophic bacterial pathogen *P. syringae*, but more susceptible to necrotrophic fungal pathogen *B. cinerea* in *Arabidopsis* (Xu et al., 2006), implying that PP2A-1 might play diverse functions when experiencing different stimuli. In our study, we confirmed that the CRISPR/Cas9-induced *pp2a-1* genome editing mutants more susceptible to *R. solani*, while the other *pp2as* were similar to wild-type plants in response to *R. solani* infection. It may be valuable to dissect the associated molecular mechanism in the future research.

As mentioned earlier, PP2As comprises three subunits (A, B, and C). In the *A. thaliana* genome, these subunits are encoded by five genes of subunit C, three genes of subunit A, and 17 genes of subunit B (Farkas et al., 2007) to establish a minimum of 255 novel forms of the molecule. Immunity in plants is influenced by subunits A and B (with scaffolding and regulatory functioning, respectively). Resistance to *P. syringae* pv. *tomato* was augmented due to a subunit B-B′θ deficient mutation (Kataya et al., 2015). AtPP2A-B′γ enhances the negatively regulated defense against *Myzus persicae* (green peach aphid) and *B. cinerea* (a necrotrophic fungus) (Trotta et al., 2011; Rasool et al., 2014). PR protein phosphorylation (PR1, PR2–PR5) is augmented by mutations in subunit *AtPP2A-B*′γ (Trotta et al., 2011). The constitutive expression of *PR1a, PR1b*, and *PR5* was induced by *NbNPP4-1* and *NbNPP4-2* silencing in *N. benthamiana* (He et al., 2004). *PR2* levels were up-regulated by *TaPP2A* silencing (Zhu et al., 2018). *PBZ1*, a *PR10* family protein accumulates in rice tissues which are in the process of cell mortality (Huang et al., 2016; Moselhy et al., 2016). In this study, *PBZ1* and *PR1b* genes were up-regulated by *R. solani* infection, suggesting that that *PBZ1* and *PR1* play a role in ShB resistance in rice. The results indicated that *R. solani-*induced *PBZ1* and *PR1b* expressions are under control of PP2A-1, suggesting that the expression of PP2A-1 might be through the activation of *PR* genes to promote rice defense.

Taken together, our findings suggest that the protein phosphatase 2A catalytic subunit, PP2A-1, regulates the defense response in rice to *R. solani* infection. This study revealed a new function of the rice *PP2A* in immune response, which provided a potential target for breeding ShB-resistant lines.

## Data Availability Statement

The original contributions presented in the study are included in the article/supplementary material, further inquiries can be directed to the corresponding authors.

## Author Contributions

QL, QM, and YX conceived and designed the studies and wrote the manuscript. QL, JC, VK, ZL, and DY collected and analyzed the data. All authors have read and approved the final version of the manuscript.

## Conflict of Interest

The authors declare that the research was conducted in the absence of any commercial or financial relationships that could be construed as a potential conflict of interest.
